# Effect of Hydrothermal Treatment on Structural and Catalytic Properties of [CTA]-MCM-41 Silica

**DOI:** 10.3390/ma11050860

**Published:** 2018-05-21

**Authors:** Iago W. Zapelini, Laura L. Silva, Dilson Cardoso

**Affiliations:** Catalysis Laboratory, Chemical Engineering Department, Federal University of São Carlos, São Carlos 13565-905, SP, Brazil; iagozapelini@gmail.com (I.W.Z.); laura.lorenadasilva@gmail.com (L.L.S.)

**Keywords:** biodiesel, condensation, mesopores, model reaction, silanol, siloxy, surfactant

## Abstract

The [CTA]-MCM-41 hybrid silica is a useful and simply prepared heterogeneous basic catalyst for the transesterification reaction. Here, the effect of hydrothermal treatment during catalyst preparation was investigated, with the aim of improving the structural stability of this catalyst during the reaction. It was observed that the hydrothermal step led to the formation of a material with a higher degree of organization and a greater wall thickness, which improved its structural stability. However, the catalyst prepared using this treatment presented lower catalytic activity, due to the presence of fewer active sites.

## 1. Introduction

Concerns related to the use of fossil fuels have led to a drive to develop sustainable alternatives, such as biofuels, based on renewable resources [1]. Biodiesel is a biofuel composed of a mixture of fatty acid esters, which is produced by the transesterification of vegetable oils or animal fats [2]. The reaction is typically performed using a short chain alcohol, such as methanol or ethanol, and is catalyzed by a basic catalyst, usually sodium methoxide (CH_3_ONa) [3]. However, the use of homogeneous catalysts makes it difficult to separate the products, because they are soluble in the glycerol produced, necessitating subsequent treatment steps that increase the cost of the production process [4].

Heterogeneous processes require fewer post-treatment steps, since separation of the catalyst is facilitated by the fact that it is not soluble in the reaction medium [4]. Basic catalysts are among those most active in the transesterification reaction, enabling high conversion rates under mild conditions [5]. These advantages, combined with the ability to recover and reuse the catalyst, make these materials highly attractive.

The oxides of alkali and alkaline earth metals are among the heterogeneous basic catalysts, which have been the most widely studied for the production of biodiesel. However, a difficulty associated with this type of catalyst is its deactivation by carbonation in the presence of CO_2_ [6]. Alternatively, mesoporous silicas containing basic sites can be used as effective basic catalysts for the transesterification reaction. These materials can be produced by functionalization of MCM-41 silica, with the creation of basic sites by means of impregnation techniques, or by condensation with silanes containing basic sites [7].

**Figure 1:**
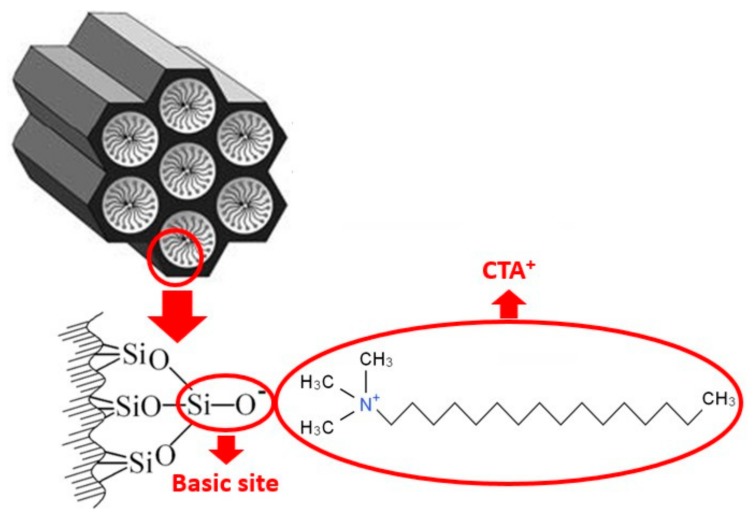
Schematic illustration of basic siloxy site generation in the MCM-41 structure by charge compensation with CTA^+^. (Modified from [9]).

Recent studies have shown that as-synthesized MCM-41 silica, which still contains the surfactant used in the synthesis within the pores, exhibits basic catalytic activity [8], due to the presence of siloxy anions (≡SiO^−^) (Figure 1) generated in the structure by charge compensation with the cations of the surfactant, usually cetyltrimethylammonium (CTA^+^) [9]. This silica can be used as a catalyst in the transesterification reaction, with high conversions achieved even under mild reaction conditions [10,11,12].

The structural stability of catalysts is an important factor to be considered, especially in applications that require severe reaction conditions or with the use of solvents that are aggressive to the catalyst structure. One way to increase the thermal and hydrothermal structural stability of catalysts is to include a hydrothermal treatment step during the preparation of these materials. In the case of the precipitation of silicas, the hydrothermal treatment step favors the silanol condensation reactions and the formation of silica, generating materials with greater mechanical stability [13].

In this work, the effect of hydrothermal treatment on the structural stability and catalytic activity of as-synthesized MCM-41 silica containing CTA^+^ cations in the pores was evaluated. These hybrid silicas were tested as basic catalysts in a model transesterification reaction between ethyl acetate and methanol.

## 2. Materials and Methods

### 2.1. Catalyst Preparation

The hybrid silicas were prepared by the method described by [14], consisting of the hydrolysis of tetraethyl orthosilicate (TEOS) in an aqueous dispersion of cetyltrimethylammonium bromide (CTABr), at a basic pH. The molar composition of the reaction mixture was 1 TEOS:12.5 NH_4_OH:0.2 CTABr:174 H_2_O. The synthesis was performed in a jacketed glass reactor coupled to a thermostatic bath, at a controlled temperature of 30 °C, under magnetic stirring. First, the CTABr (Sigma-Aldrich, St. Louis, MO, USA, 98%) was dissolved in the mixture of distilled water and ammonium hydroxide (Synth, 28%). After complete solubilization of the surfactant, TEOS (Sigma-Aldrich, St. Louis, MO, USA, 98%) was slowly added dropwise, and the mixture was then kept under stirring for 2 h. The solid material obtained at the end of this procedure was filtered, washed until reaching pH 7, and dried in an oven at 50 °C for 12 h. The silica obtained at ambient temperature was denoted M-RT.

To obtain the hydrothermally-treated sample, after precipitation of the silica during 2 h, the reaction mixture was transferred to a Teflon reactor and heated in an oven at 100 °C for 24 h. The solid was then filtered, washed, and dried. The silica obtained was denoted M-HT.

### 2.2. Characterization of the Catalysts

Small-angle X-ray scattering (SAXS) analysis of a 0.064 mol·L^−1^ aqueous dispersion of CTABr was performed at the SAXS2 beamline of the National Synchrotron Light Laboratory (LNLS) in Campinas (LNLS, São Paulo State, Brazil). Scattering measurements at angles between 0.1 and 10° were performed using a light beam with wavelength (λ) of 0.15498 nm and a detector-sample distance of 562.5359 mm, obtaining a curve of the scattering intensity as a function of the scattering vector (q).

The structures of the synthesized silicas were characterized by powder X-ray diffraction (XRD), using a Miniflex 600 (Rigaku, Tokyo, Japan) diffractometer operated with Cu Kα radiation (λCu-Kα = 0.15418 nm), with scanning of 2θ angles between 1.5° and 7.5°, at a goniometer speed of 2°·min^−1^. The interplanar distances (d) were calculated from the diffraction peaks using the Bragg equation (Equation (1)).

D = λ_(Cu-Kα)_/2sinθ, (1)

The pore diameters, d_p_, were calculated using the BJH (Barrett-Joyner-Halenda) method, based on the equilibrium phase transition in cylindrical pores [15], the were applied to nitrogen desorption branch of adsorption–desorption isotherms acquired at 77 K using a ASAP 2020 instrument (Micrometrics, Norcross, USA). The samples were previously calcined at 550 °C for 4 h to remove the surfactant. Before the measurements, the samples were treated under a vacuum at 250 °C for 4 h to remove physisorbed water.

**Figure 2:**
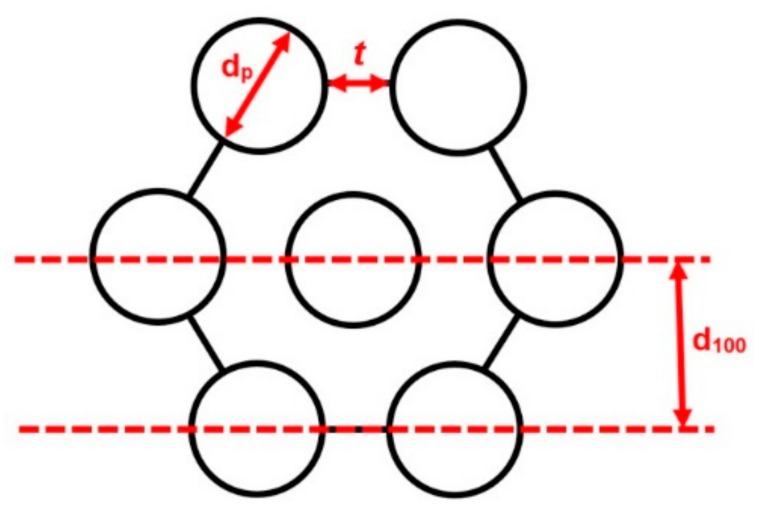
Schematic illustration of the hexagonal arrangement of the MCM-41 pores, indicating the (100) plane interplanar distance (d_100_), the pore diameter (d_p_), and the wall thickness (*t*).

For each calcined silica, the pore diameter and the d_100_ interplanar distance, corresponding to the (100) plane (Figure 2), were used to calculate the wall thickness, *t*, according to Equation (2).

(2) $$d_{100}=0.5\surd 3(d_{p}+t),$$

Thermogravimetry (TG) was used to determine the cation content of each synthesized material, together with the amount of structural silanol (see the Supplementary Material). The analyses were performed using a Model SDT Q600 thermobalance (TA Instruments, New Castle, USA). An approximately 0.01 g mass of the sample was heated from 30 to 830 °C in an alumina sample holder, using a heating rate of 10 °C·min^−1^, in an oxidizing atmosphere of synthetic air supplied at a flow rate of 30 mL·min^−1^.

The basicity of the ≡SiO^−^ sites of the as-synthesized silicas was characterized by O1s X-ray photoelectron spectroscopy (XPS). The spectra were acquired using a ESCA+ EAC 2000 instrument coupled to a high-performance hemispherical analyzer (Scienta Omicron, Taunusstein, Germany), with excitation using Al Kα radiation (hν = 1486.6 eV). The contributions of the ≡SiO^−^CTA^+^, ≡SiOSiO≡, and ≡SiOH species were calculated by signal deconvolution [16].

Scanning electron microscopy (SEM) images were obtained after the solids had been dispersed in acetone and submitted to ultrasonic de-agglomeration. Aliquots of the suspensions were deposited on polished aluminum sample holders, followed by analysis using a Magellan 400 L (FEI, Hillsboro USA) microscope operating at 25 kV.

### 2.3. Catalytic Tests

**Figure 3:**
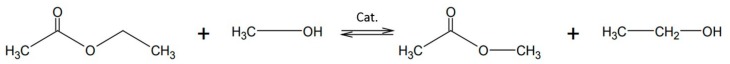
Ethyl acetate methanolysis as a model transesterification reaction.

The hybrid silicas were tested using the methanolysis of ethyl acetate as a model transesterification reaction (Figure 3). The reactions were performed in a jacketed glass batch reactor, under stirring, at a controlled temperature of 30 °C (maintained using a water bath). A molar ratio of methanol to ethyl acetate of 6:1 was used, with 4% (by mass) of catalyst.

The reaction was monitored for 60 min, with aliquots collected at predetermined times for immediate analysis using a GC 2010 (Shimadzu, Tokyo, Japan) gas chromatograph fitted with a flame ionization detector (FID). The sample aliquots were removed using a polyethylene syringe containing a PVDA microfilter (Agilent, Santa Clara, USA). The products and reagents were separated on an RTX-1 column (30 m × 0.25 mm × 0.25 μm) with a polyethylene glycol stationary phase, using helium as the carrier gas.

The conversion rates obtained for the catalysts were calculated by fitting a hyperbolic function, which intercepts the (0;0) point to the experimental data for the conversion of ethyl acetate as a function of the reaction time, taking zero time as a reference for determination of the turnover frequency (TOF_0_, see the Supplementary Material).

## 3. Results and Discussion

**Figure 4:**
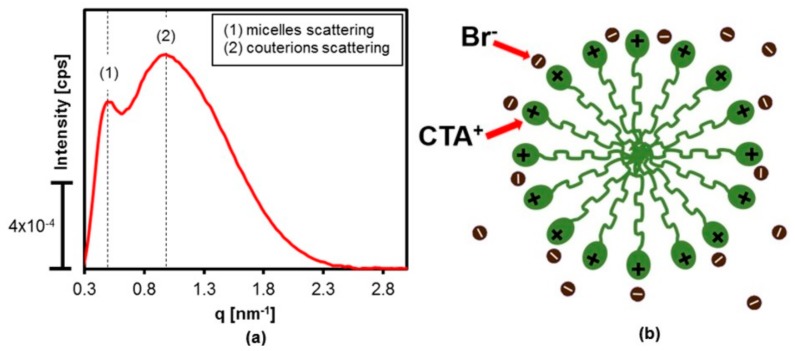
(**a**) SAXS curve of the aqueous 0.064 mol·L^−1^ CTABr dispersion used for [CTA]-MCM-41 preparation; (**b**) schematic representation of the CTABr micelles.

Figure 4a shows the SAXS curve for the 0.064 mol·L^−1^ dispersion of CTABr in water. According to the literature, the scattering curve for this dispersion presents two correlation peaks. The first, at around 0.5 nm^−1^, corresponds to scattering by the nuclei of the micelles formed by the hydrophobic CTA^+^ tails of the surfactant. The second, at around 1 nm^−1^, corresponds to the X-ray scattering caused by the layer of bromide counterions organized around the heads of the cationic micelles [17]. It could be inferred from the presence of the two scattering peaks in the SAXS curve for the aqueous dispersion of the surfactant that it was composed of CTABr micelles, which provided a template for formation of the mesoporous MCM-41 silica [18].

**Figure 5:**
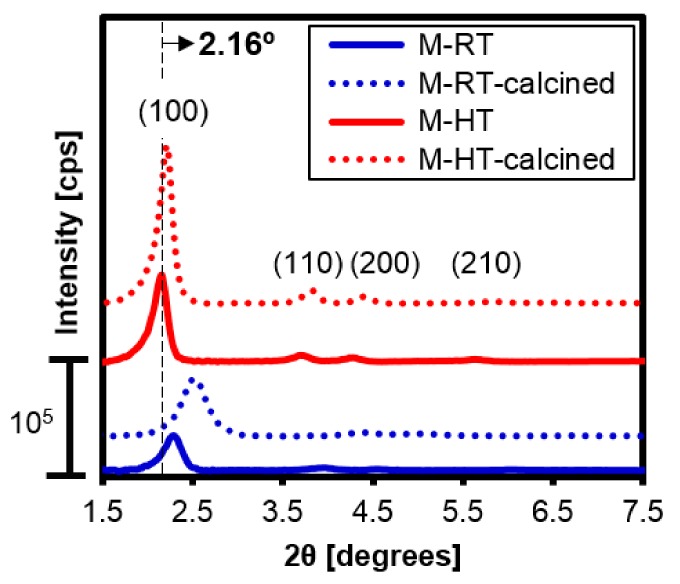
XRD patterns of the as-synthesized and calcined silicas.

The X-ray diffractograms of the as-synthesized silicas are shown in Figure 5 (solid lines). The ratios obtained between the interplanar distances (Table 1) confirmed formation of the MCM-41 structure. The silica prepared using hydrothermal treatment (M-HT) presented more intense diffraction peaks, with the relative intensity of the less-organized sample (M-RT) being about 40% of the value obtained for the more organized material, based on the peak corresponding to the (100) plane (Table 1).

The X-ray diffractograms (Figure 5) showed that the calcined hybrid silicas maintained the MCM-41 type structure, with the diffraction peaks becoming more intense, probably due to loss of the organic cations. In addition, the diffraction peaks shifted to larger angles, which, according to Bragg’s law, resulted from contraction of the corresponding interplanar distances. The greatest displacement observed for M-RT-calcined silica was due to the higher amount of CTA^+^ present in its structure, which after removal during calcination causes the contraction of the interplanar distance. The CTA^+^ amounts will be presented in thermal analysis.

The hydrothermal treatment resulted in the diffraction peaks shifting to smaller angles. According to Bragg’s law, this was due to expansion of the distance between the corresponding planes. As shown in Figure 2, the d_100_ interplanar distance is a direct function of the sum of the silica wall thickness (*t*) and the pore diameter (d_p_) of the material (Equation (2)). Hence, an increase of the interplanar distance can be a result of both a greater pore diameter and thickening of the silica wall.

**Figure 6:**
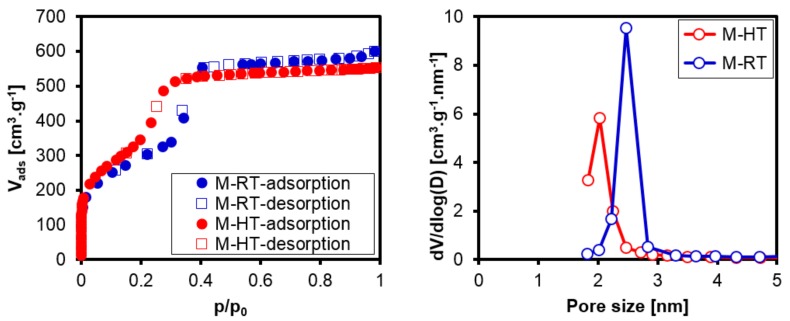
(**a**) Nitrogen adsorption and desorption isotherms (obtained at 77 K) for the calcined silicas; (**b**) the pore diameter distribution obtained using the BJH method.

The nitrogen adsorption and desorption isotherms obtained at 77 K (Figure 6a) were type IV, typical of mesoporous materials [20]. An increase of the adsorbed volume at relative pressures between 0.2 and 0.5, with the presence of an inflection, corresponded to the capillary condensation of N_2_ within the mesopores, as typically observed for materials of the M41S family [21].

The upwards shift of the slope for filling of the mesopores at lower relative pressures, observed for sample M-HT, was a result of the decreased pore diameter. Table 2 shows the pore diameters of the calcined silicas, obtained from the nitrogen physisorption isotherms using the BJH method, together with the wall thicknesses calculated using Equation (2). The hydrothermal treatment led to increased wall thickness of the silica, in agreement with previous studies [13,20]. The absolute values of pore diameters have to be treated with caution, because in general, the BJH method underestimates the pore diameter of mesoporous materials [22].

**Figure 7:**
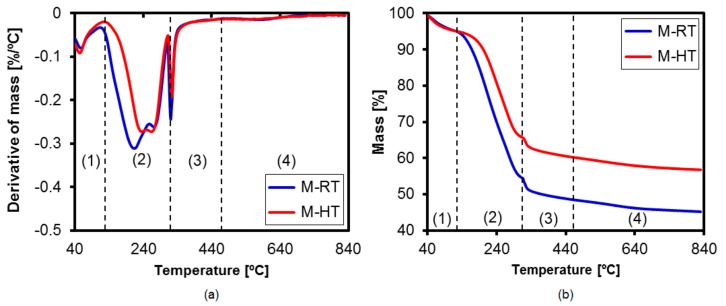
(**a**) DTG (derivative of mass) curves; (**b**) thermograms of the as-synthesized silicas. The different regions indicated correspond to (1) water desorption, (2) surfactant degradation by Hoffman reactions, (3) degradation of residual products, and (4) condensation of silanol groups.

According to [23], the [CTA]-MCM-41 silica presents four regions of mass loss during heating. The first corresponds to the elimination of physically adsorbed water. The second region, which accounts for the greatest mass loss of the material, is due to decomposition of the surfactant by means of Hoffman reactions. In the third region, residual amines are desorbed or decomposed. Finally, there is dehydroxylation of structural silanol groups of the material. The DTG curves for both silicas (Figure 7a) revealed these regions of mass loss. In the case of the silica prepared with hydrothermal treatment, the surfactant decomposition occurred at higher temperatures, indicative of the greater difficulty of removal of the CTA^+^ cations from the more organized material. This suggested that the cations were mainly located within the pores, and to a lesser extent on the exterior of the silica, compared to the silica that was not submitted to heat treatment.

The thermograms (Figure 7b) revealed greater mass loss of the [CTA]-MCM-41 prepared at ambient temperature, compared to the hydrothermally-treated material, resulting in a lower mass percentage of silica. The curves were used to calculate the mass losses in each region (Table 3). The CTA^+^ contents of the samples were obtained from the mass losses in regions (2) and (3), corresponding to degradation of the surfactant and its residual products. The silica prepared at ambient temperature presented an organic cation content that was approximately 1.7-fold higher than for the [CTA]-MCM-41 prepared using hydrothermal treatment. This could be explained by the greater condensation of silica at high temperatures [13], resulting in a decrease of the overall quantity of ≡SiO^−^ ions in the silica prepared at higher temperature.

**Figure 8:**
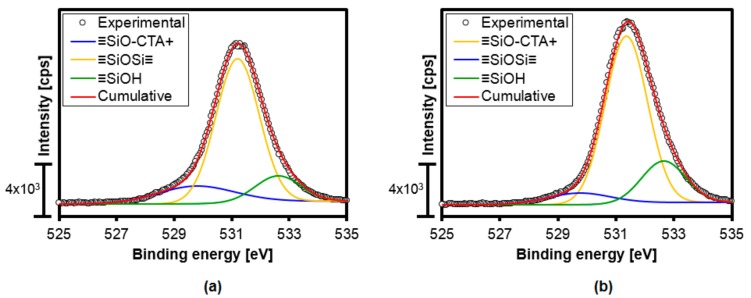
O1s XPS spectra of the hybrid silicas M-RT (**a**) and M-HT (**b**), and deconvolution of the signals.

The O1s XPS results were in agreement with the thermogravimetry data. Deconvolution of the XPS signal (Figure 8) enabled determination of the contributions of the signals for the ≡SiOH, ≡SiO^−^CTA^+^, and ≡SiOSi≡ species, with good fits obtained (R^2^ > 0.99). The results for each integrated signal are shown in Table 4. The M-RT silica presented a higher content of ≡SiO^−^CTA^+^, compared to the M-HT material, indicating that the condensation of silica at high temperatures led to fewer catalytic sites.

**Figure 9:**
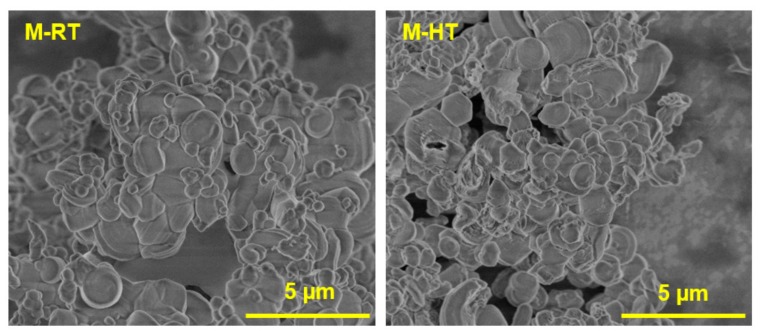
SEM images of the [CTA]-MCM-41 hybrid silicas.

The SEM micrographs of the as-synthesized hybrid silicas (Figure 9) showed that the two synthesis conditions resulted in rounded silica particles with irregular dimensions, which were of similar sizes for the two materials.

**Figure 10:**
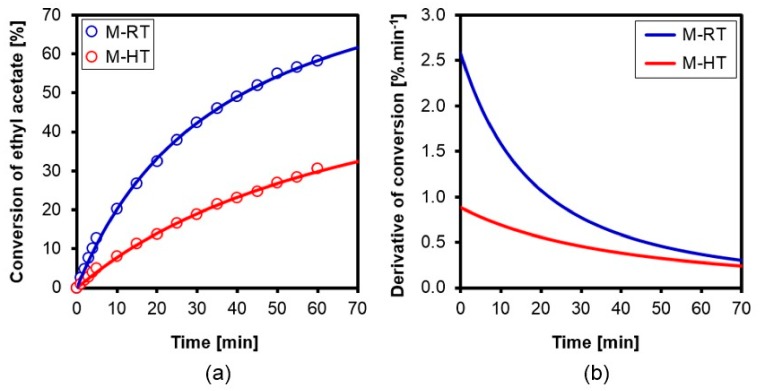
Conversion (**a**) and derivative conversion (**b**) curves for the [CTA]-MCM-41 hybrid silicas used in ethyl acetate methanolysis.

The hybrid silicas were evaluated as basic catalysts in the model transesterification reaction (Figure 3). Due to the presence of the charge-compensating CTA^+^ cations occluded in the pores or dispersed on the surface of the as-synthesized silicas, these materials exhibited basic catalytic activity generated by the ≡SiO^−^ anions [9]. Figure 10 shows the ethyl acetate conversion curves for the two catalysts, according to time, from which it can be seen that higher conversion was always achieved for the material prepared without hydrothermal treatment.

Hyperbolic fitting resulted in R^2^ > 0.99 for both curves (Table 5), with higher calculated reaction rates (Figure 10) for the catalyst prepared without hydrothermal treatment. This could be explained by the fact that this material possessed a greater number of catalytic sites, generated by the higher number of cations, as shown in Table 3.

The specific conversions at the beginning of the reaction, given by the TOF_0_ values (Table 5), showed that the silica without hydrothermal treatment was more effective in the conversion of ethyl acetate to methyl acetate, with a turnover frequency (TOF_0_) about 2.2-fold higher than that of the silica prepared at an elevated temperature.

There are two factors that can influence the TOF_0_ of a catalyst—namely, the strength of the active sites and their accessibility. In the present case, the catalytic sites presented the same strength for the two catalysts, as shown by the O1s XPS results (Table 4), since they corresponded to the ≡SiO^−^ sites compensating CTA^+^.

In terms of site accessibility, the pores of both hybrid silica catalysts were filled with surfactant, so the catalysis necessarily occurred at the sites located in the pore mouth or in the external area of the silica particles [24], and was mainly dependent on the particle size and the quantity of sites on the external surface of the material. The micrographs (Figure 9) showed that there was no significant difference between the particle sizes of the two catalysts, so there was only the effect of the quantity of surface sites present on the silicas.

In order to investigate this effect, analysis was made of the shift of the mass loss band associated with decomposition of the surfactant (Figure 7a), which occurred at higher temperatures for the M-HT catalyst. This behavior can be explained by the smaller amount of surfactant on the surface of the silica, leading to a decrease of TOF_0_, because the sites were less accessible to the reagents. In the case of the M-RT silica, there was a greater amount of surfactant on the external surfaces of the silica particles, generating sites that were more accessible and increasing the specific conversion for this catalyst.

**Figure 11:**
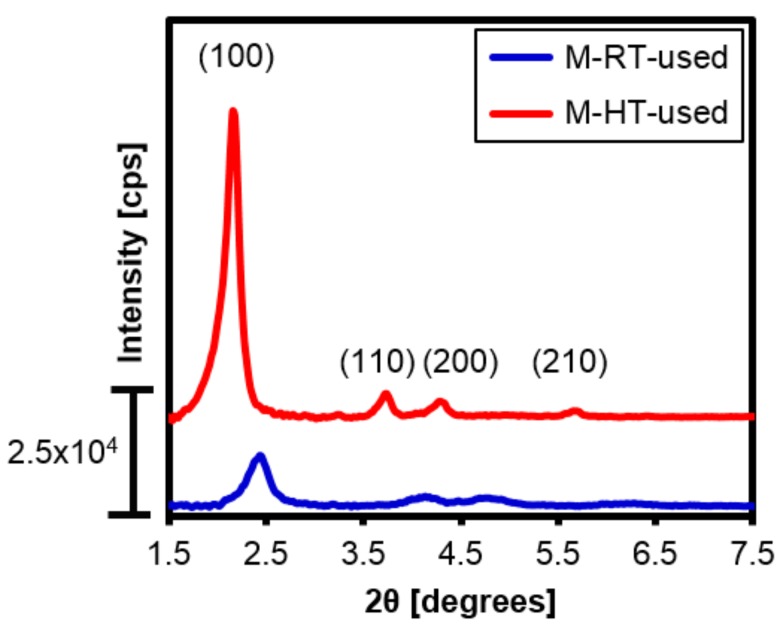
X-ray diffractograms of the [CTA]-MCM-41 hybrids after use in the transesterification reaction.

The X-ray diffractograms of the silicas recovered at the end of the transesterification reaction (Figure 11) showed that the characteristic diffraction pattern of MCM-41 was maintained. A relative intensity of 13% was obtained for the (100) peak of the used M-RT catalyst, compared to the M-HT silica, corresponding to a decrease of 71% relative to the peak obtained for the material before use. The M-HT silica used presented a relative peak intensity of 81%, compared to the signal obtained before use. These results showed that the hydrothermal treatment led to a catalyst with greater structural stability when used in the transesterification reaction.

The decreases of the diffraction peaks for both silicas could have been due to the reaction of methanol with the silica surface, forming methoxysilane groups (≡SiO-CH_3_) [25] that reduced the degree of organization of the material. The higher stability of the M-HT sample during the catalysis could be attributed to the greater wall thickness (Table 2), which increased its mechanical resistance [13]. In addition, the higher number of ≡SiOSi≡ groups in its structure (Table 3 and Table 4) could act to increase the stability of the wall.

## 4. Conclusions

The [CTA]-MCM-41 hybrid silica presented catalytic activity in the transesterification reaction, even under mild conditions. The addition of a hydrothermal treatment step in the preparation of this catalyst led to improved structural stability, due to the greater thickness of the silica wall. However, the use of this additional step resulted in formation of fewer catalytic sites, hence reducing the activity in the transesterification reaction.

## Figures and Tables

**Table 1:** Relative intensities (RI) of the peaks corresponding to the (100) planes of the as-synthesized silicas, and interplanar distance ratios.

Catalyst	RI (%)	d_100_/d_110_	d_100_/d_200_	d_100_/d_210_
M-RT	40	1.72	1.98	2.65
M-HT	100	1.71	1.98	2.61
Theoretical [19]	-	1.73	2.00	2.60

**Table 2:** Interplanar distances of the (100) planes (d_100_), pore diameters (d_p_), and wall thicknesses (*t*) of the calcined silicas.

Catalyst	d_100_ (nm)	d_p_ (nm)	*t* (nm)
M-RT-calcined	3.5	2.5	1.5
M-HT-calcined	4.0	2.0	2.6

**Table 3:** Mass loss calculations for regions (1), (2), (3), and (4), and contents of CTA^+^ groups in the [CTA]-MCM-41 hybrid silicas.

Catalyst	SiO_2_ (% by Mass)	Mass Loss (%)				[CTA^+^]/[SiO_2_]	[≡SiOH]/[SiO_2_]
		1	2	3	4		
M-RT	45.19	4.82	40.35	6.42	3.21	0.22	0.26
M-HT	56.77	4.99	29.08	6.25	2.91	0.13	0.21

**Table 4:** Contributions of O1s species in the XPS signals of the hybrid silicas.

Catalyst	O1s Binding Energy (eV)			Area (%)		
	≡Si-O^−^CTA^+^	≡SiOSi≡	≡Si-OH	≡Si-O^−^CTA^+^	≡SiOSi ≡	≡Si-OH
M-RT	529.76	531.19	532.60	15.67	71.02	13.31
M-HT	529.76	531.36	532.65	7.62	73.51	18.87

**Table 5:** Parameters of the hyperbolic fits, y = ax/(x + b), for ethyl acetate (A) conversion (*y*) as a function of time (*x*), and the initial turnover frequency (TOF_0_) values of the catalysts.

Catalyst	a	b	R^2^	(dX_A_/dt)_0_(%·min^−1^)	TOF_0_ (mol A.mol site^−1^·min^−1^)
M-RT	93.64	36.32	0.999	2.58	139
M-HT	68.35	77.22	0.998	0.89	64

## Supplementary Materials

The following are available online at http://www.mdpi.com/1996-1944/11/5/860/s1.
