# Supplementary material for: Effect of Hydrothermal Treatment on Structural and Catalytic Properties of [CTA]-MCM-41 Silica
Source: Materials (Basel). 2018 May 21;11(5):860. doi: 10.3390/ma11050860 (PMC5978237; doi:10.3390/ma11050860)
Supplement: Supplementary file 1 [file materials-11-00860-s001.pdf]

materials

MDPI

Supplementary material

Effect of Hydrothermal Treatment on Structural and Catalytic Properties of [CTA]-MCM-41 Silica

Iago W. Zapelini, Laura L. Silva and Dilson Cardoso *

Received: date; Accepted: date; Published: date

Calculation of [CTA⁺]/[SiO₂] ratio in hybrid silicas

In order to obtain the content of catalytic sites ($\equiv SiO^-$) using thermogravimetric analysis, the molar ratio of $[CTA^+]/[SiO_2]$ were calculated based on CTA^+ decomposition observed by DTG curves, according to the Equation S1:

$$\frac{[\mathrm{CTA}^+]}{[\mathrm{Si}O_2]} = \frac{\frac{m_{2-3}}{MM_{CTA^+}}}{\frac{m_{SiO_2}}{MM_{SiO_2}}} \qquad \text{(S1)}$$

where $m_{2\text{-}3}$ is the mass loss in regions (2) and (3) of the thermograms, MM_{CTA+} is the molar mass of CTA^+ cation, m_{SiO2} is the remaining mass after thermal analysis and MM_{SiO2} is the molar mass of the silica.

Calculation of [≡SiOH]/[SiO₂] ratio in hybrid silicas

The hybrid silicas are calcined during thermogravimetric analysis (heating from 30 to 830 °C). During this process, two $\equiv SiO^- CTA^+$ groups are converted to one ≡SiOH group by the surfactant removal and elimination of one molecule of H_2O. The mass loss in dihydroxylation region corresponds to water exit, generated by ≡SiOH condensation, including structural silanol (formed during the silica preparation) and silanol groups formed by CTA^+ removal. Structural silanol were calculated by Equation (S2):

$$\frac{[\mathrm{CTA}^+]}{[\mathrm{Si}O_2]} = \frac{\frac{m_4}{MM_{H_2O}} - 0.5\frac{m_{2-3}}{MM_{CTA^+}}}{\frac{m_{SiO_2}}{MM_{SiO_2}}} \qquad \text{(S2)}$$

where m_4 is the mass loss in the dihydroxylation region and MM_{H2O} is the molar mass of water.

Calculation of TOF_0

Using the values of derivative curves in 0 min $(dX_A/dt)_0$, turnover frequencies (TOF_0) were calculated using the $[CTA^+]/[SiO_2]$ ratios, considering the molar content of catalytic sites equal to this ratio. TOF_0 were obtained using the equation below:

$$TOF_0 = \frac{MM_{SiO_2} C_{A0} V (dX_A/dt)_0}{\% SiO_2 m_{cat} \frac{[\mathrm{CTA}^+]}{[\mathrm{Si}O_2]}} \tag{S3}$$

where C_{A0} (2.94 mol.L^{-1}) is the initial concentration of ethyl acetate, m_{cat} (0.6667 g) is the mass of catalyst used in each experiment and V (20.2 mL) is the reactional volume.
